# The prognostic value of methylated ctDNA, soluble PD-L1, and NK-cell activity on the risk of relapse after curative radiotherapy of non-small cell lung cancer

**DOI:** 10.1007/s12094-026-04317-5

**Published:** 2026-03-16

**Authors:** Thomas Leth Fink, Rikke Fredslund Andersen, Cecilie Mondrup Jacobsen, Line Nederby, Mads Malik Aagaard Jørgensen, Charlotte Kristiansen, Torben Schjødt Hansen, Sara Witting Christensen Wen, Christa Haugaard Nyhus, Rune Slot Thing, Signe Timm, Torben Frøstrup Hansen

**Affiliations:** 1 Department of Oncology, Lillebaelt Hospital, Vejle, University Hospital of Southern Denmark, 7100 Vejle, Denmark; 2 Institute for Regional Health Research, University of Southern Denmark, Odense M, Denmark; 3 Department of Biochemistry and Immunology, Lillebaelt Hospital, Vejle, University Hospital of Southern Denmark, 7100 Vejle, Denmark; 4 Department of Clinical Genetics, Lillebaelt Hospital, Vejle, University Hospital of Southern Denmark, 7100 Vejle, Denmark

**Keywords:** Lung cancer, Radiotherapy, Liquid biopsy, Natural killer-cell activity, Soluble PD-L1, Methylated ctDNA

## Abstract

**Introduction:**

Despite receiving curative radiotherapy (RT) for localized lung cancer, relapse may occur within the first year, and 5-year survival is low. In this study, we evaluated the prognostic ability of the combination of Natural Killer-cell activity (NKA), soluble programmed death-ligand 1 (sPD-L1), and circulating tumor DNA (ctDNA) to predict a relapse within 12-month follow-up.

**Materials and methods:**

Sixty-eight patients had blood samples analyzed from baseline, the first follow-up visit, and 6 and 9 months after treatment. NKA was measured with the NKVue® assay, sPD-L1 was measured with the Quantikine ELISA kit, and ctDNA was measured using in-house developed tumor-agnostic droplet digital polymerase chain reaction (ddPCR) assays for testing methylated loci near the genes *HOXA9*, *TFAP2B*, *MCIDAS,* and *SP9*. Chi^2^ statistics or Fisher’s exact test was used for individual markers, and ROC analyses were used for the combined markers.

**Results:**

Baseline reduced NKA was significantly associated with experiencing a relapse within 12-month follow-up (*p* = 0.01). Having a positive baseline ctDNA was also significantly associated with experiencing a relapse within 12-month follow-up (*p* = 0.03). The combination of all four ctDNA markers, sPD-L1, and NKA had an area under the curve of 0.86 (95% CI 0.74–0.98) for predicting a relapse within 12 months of follow-up.

**Conclusion:**

Findings from this study suggest that a combination of all six biomarkers measured at baseline may help predict the risk of relapse following curative RT for lung cancer. Larger studies with longer follow-up are needed to further verify the results.

**Supplementary Information:**

The online version contains supplementary material available at 10.1007/s12094-026-04317-5.

## Introduction

Curative radiotherapy (RT) is offered to patients with early stage or locoregional non-small cell lung cancer (NSCLC) not fit or eligible for, or not wanting surgery, either as stereotactic body radiation therapy (SBRT) or as long-course RT. Despite the curative intent, relapse of lung cancer is seen within the first year, and the 5-year survival in Denmark for all lung cancer stages is only around 27% [1]. Better means of providing a reliable prognosis for these patients could help select the patients needing extra treatment, and earlier detection of relapse could enable more patients to receive a curative treatment for their relapse.

A patient with a solitary lung tumor of up to 5 cm and World Health Organization performance status (WHO PS) 3 or better can be offered SBRT, with a dose/fractionation of 66 Gray (Gy) in three fractions being the standard in Denmark [2]. This treatment offers 90% or better local control after 2 years and only a few side effects; however, it requires that the tumor has an appropriate distance from organs at risk [3]. Patients with larger tumors and/or locoregional lymph-node involvement can be offered long-course RT, with a dose/fractionation of 66 Gy in 33 fractions being the standard in Denmark [2]. Concomitant chemotherapy with a platinum-based drug and/or vinorelbine is offered to most patients (PS 0–2) along with the long-course RT. Patients with a histologic PD-L1 expression > 1% and PS 0–1 are also offered adjuvant immune checkpoint inhibitor treatment with Durvalumab for 1 year following the completion of chemoradiotherapy. Chemoradiotherapy has a poorer rate of local control compared to SBRT, and relapses frequently occur, often during the first few years after the treatment [4].

Blood-based biomarkers are obtained through minimally invasive procedures, are readily available, and have been studied intensively in recent years. Previous studies have indicated that such markers may predict the effect of treatment or indicate better/worse prognosis for patients with lung cancer [5]. The molecular biology technique droplet digital polymerase chain reaction (ddPCR) uses partitioning of samples into thousands of nanoliter-sized droplets. The polymerase chain reaction takes place in each of these droplets, allowing detection of molecular targets in very low abundance with very high sensitivity [6]. With the use of newer multiplex systems, several targets can be analyzed simultaneously, allowing faster analyses and higher sensitivity for detecting molecular targets [7]. Methylated Homeobox A9 (*HOXA9*) circulating tumor DNA (ctDNA) measured with ddPCR has been demonstrated as a valuable prognostic marker in patients with advanced NSCLC [8], but it is not known whether *HOXA9* alone or in combination with other tumor-agnostic methylated ctDNA markers offers any prognostic value in curatively irradiated patients with NSCLC.

Soluble Programmed Death-Ligand 1 (sPD-L1) has been tested as a prognostic biomarker in patients with NSCLC, and the previous results indicate a correlation between the level of sPD-L1 and risk of recurrence and overall survival (OS) [9, 10]. It is not known whether combining sPD-L1 with other biomarkers can enhance the prognostic value.

NK-cell activity (NKA) as measured with the NKVue® assay using interferon-γ (IFN-γ) as a surrogate for NKA has shown reliable and reproducible results in an earlier study from our group [11]. Patients showing an abnormally low level of NKA during palliative systemic therapy have been shown to have a poorer prognosis across different cancer types [12]. It is not known whether the baseline NKA itself may hold prognostic value in NSCLC.

### Aim

The aim of this study was to evaluate the prognostic and relapse-detecting value of the biomarkers NKA, sPD-L1, and methylated ctDNA measured at baseline and during 12 months of follow-up in NSCLC patients treated with curative RT.

## Materials and methods

We follow the REMARK guideline for the reporting of tumor marker prognostic studies [13].

### Patients

This prospective biomarker study was conducted at the Department of Oncology, Lillebaelt Hospital, University Hospital of Southern Denmark, Vejle, Denmark.

All patients were prospectively included and gave oral and written consent to participate. The study conforms to the Declaration of Helsinki and Danish data protection legislation. The study protocol was approved by the Regional Health Research Ethics Committee of Southern Denmark (ID-number S-20180123).

Patients with primary NSCLC with or without locoregional lymph-node involvement planned for curative RT were offered enrollment in the study (corresponding to TNM 8th edition stage Ia–IIIc) [14].

#### Inclusion criteria

Stereotactic or conventional RT for primary NSCLC with curative intent and concurrent chemotherapy if indicated, age ≥ 18 years, WHO performance status 0–2, written and orally informed consent, consent to translational research and biobank.

#### Exclusion criteria

Distant metastases, severe comorbidity making the patient unlikely to complete the planned 2-year follow-up period, other malignant disease within 5 years prior to study enrollment, except basocellular or squamous skin cancer and carcinoma in situ cervicis uteri.

To increase the number of patients in the analyses, we included ten comparable patients from another cohort, which had blood sampled twice during their diagnostic work-up and curative RT treatment. These blood samples were collected between June 2022 and December 2023. These ten patients were all diagnosed with NSCLC, and six of these patients received SBRT, while four patients received long-course RT.

Permission to use the blood sampled from these patients for this study was obtained as an amendment to the original protocol accepted by the Research Ethics Committee of Southern Denmark (S-20180123). This other cohort did not include NKVue blood sampling, so the added ten patients could not contribute data to NKA analyses.

### Treatment

All patients received standard treatment according to Danish guidelines [2, 15].

The RT was delivered either as SBRT with 3–5 fractions (*n* = 33) or as long-course chemoradiotherapy with 24–33 fractions (66 Gy in 33 fractions (*n* = 26), 66–95 Gy in 33 fractions inhomogeneously escalated according to the NARLAL2 trial (*n* = 3) [16], or 50–66 Gy in 24 fractions inhomogeneously escalated according to the HERAN trial (*n* = 6) [17]).

### Follow-up

The first follow-up visit was made five weeks after the completion of RT. Subsequent follow-up visits were planned with 3-month intervals for the first two years and then 6-month intervals for the next three years for a total of 5-year follow-up, as recommended by Danish guidelines [2]. A CT scan of the thorax and abdomen preceded each follow-up visit. In this paper, we only provide information on 12 months of follow-up, as many patients have not yet completed 2-year follow-up.

### Blood sampling and storage

Blood sampling was performed before the start of radiation treatment, at each cycle of chemotherapy, at the end of radiation treatment, and at each follow-up visit for up to 2 years after treatment, or until relapse. At each blood sampling, three 9 mL venous blood samples were collected in EDTA tubes for plasma, and a 9 mL sample was collected in a clot activator tube for serum. The blood collection tubes were centrifuged at 2000 g for 10 min within 4 h and stored at − 80 °C until analysis. Storage time ranged from a few months to 4 years. One milliliter of venous blood was also sampled into a Promoca™-containing NK Vue® tube for analysis of NKA and placed in an incubator at 37 °C within 15 min of the blood sampling. After 20–24 h of incubation, the plasma was collected and stored at − 80 °C for a maximum of 54 days before analysis.

### Analysis of methylated ctDNA

Four milliliters of plasma was thawed at room temperature and centrifuged at 10,000 g for 10 min. ~ 9000 copies/mL of an exogenous spike-in DNA fragment (*CPP1*) were added to the plasma to evaluate DNA extraction efficiency [18]. DNA was extracted using the DSP Circulating DNA kit (Qiagen, Hilden, Germany) on the QiaSymphony SP instrument (Qiagen, Hilden, Germany) according to the manufacturer’s instructions. Extraction efficiency was determined with a ddPCR assay targeting *CPP1* and calculating the percentage of recovered *CPP1* copies/mL. Total cfDNA concentration and contamination with high-molecular-weight DNA were determined with ddPCR assays amplifying a 65 bp and 250 bp region of the *EMC7* gene [19]. Potential contamination with lymphocyte DNA was evaluated with an immunoglobulin-specific ddPCR assay [18]. The extracted DNA was concentrated to 20 µL with an Amicon Ultra-0.5 centrifugal filter unit (Merck, Darmstadt, Germany) and bisulfite converted using the EZ DNA methylation lightning kit (Zymo Research, Irvine, CA, USA) according to the manufacturer’s instructions. Water was used as a non-template control, leukocyte DNA from healthy donors (20 µL corresponding to ~ 20 ng) as a non-cancer control, and Universal Methylated DNA Standard (Zymo Research, Irvine, CA, USA) as a positive control. All the controls were analyzed in parallel with the patient samples, and all analyses were blinded to the clinical endpoints.

The analyses of ctDNA methylation were performed in duplicate using the Bio-Rad QX600 Droplet Digital PCR Systems (Bio-Rad, Hercules, CA, USA). Droplets were generated using an Automated Droplet Generator (Bio-Rad). The PCR was performed on a Veriti™ Thermal Cycler (Applied Biosystems, Waltham, MA, USA) with the following PCR conditions: 95 °C for 10 min, 44 cycles of 95 °C for 15 s and 56 °C for 1 min, and 98 °C for 10 min using 2 °C/s ramp rate. All data were analyzed with QXManager Software 2.0 (Bio-Rad). Four methylated loci near the genes *HOXA9*, *MCIDAS*, *TFAP2B*, and *SP9* were included in the multiplex. These methylated loci were selected based on previous studies (*HOXA9*) and data from several databases on candidate tumor-agnostic lung cancer methylated areas. These databases include The Cancer Genome Atlas Program (TCGA) and Gene Expression Omnibus (GEO) [20]. The selection process and validation of candidate targets is described in a recent paper from our group [21]. The analyses were developed and validated in-house in our lab.

The four methylated ctDNA markers were previously tested in a cohort of 40 patients from the lung clinic at Vejle Hospital, where patients with benign lung disease, such as chronic obstructive pulmonary disease or asthma, are treated. These 40 patients were not known to have any malignant disease at the time of blood sampling. The limit of blank (LoB) for each marker was established following the measurements from these 40 non-cancerous subjects, and using a set ≥ 95% specificity, the LoB was set at two positive droplets for the *HOXA9*, *MCIDAS,* and *TFAP2B* markers and at three positive droplets for the *SP9* marker. In addition, a sample must have at least two markers above LoB to be classified as positive.

### Analysis of soluble PD-L1

sPD-L1 was analyzed in all samples using the Human/Cynomolgus Monkey PD-L1/B7-H1 Quantikine ELISA kit in a 1:2 dilution (R&D Systems, Minneapolis, Minnesota, USA) according to the manufacturer’s recommendations. As controls, four serum samples were analyzed in duplicates. The in-house intra-assay variation was < 13%, and the inter-assay variation was < 11%. The analyses were blinded to the clinical endpoints.

### Analysis of NK-cell activity

NK-cell activity was measured utilizing the level of IFN-γ as a surrogate with the NK Vue® Kit (NKMAX, Seongnam-si, South Korea). This technique has previously been reported by our group [11, 12]. Frozen plasma samples were thawed at room temperature and centrifuged at 11,500 g for 1 min before being analyzed using the NK Vue® enzyme-linked immunosorbent assay (ELISA) (NKMAX, Seongnam-si, South Korea). The manufacturer’s instructions were followed in all steps of the procedure. In-house measurements of the ELISA showed an intra-assay coefficient of variation < 10% and an inter-assay coefficient of variation < 14%. A level of < 250 pg/mL IFN-γ was considered abnormal as recommended by the manufacturer. The analyses were blinded to the clinical endpoints.

### Statistics

Continuous variables were reported as means with standard deviation, if normally distributed (tested with visual inspection of QQ-plots), and as median with range and/or interquartile range (IQR) if non-normally distributed.

Associations between biomarker values (positive/negative) and 12-month relapse were calculated with Chi-square statistics, or with Fisher’s exact test if any observed frequency was below *N* = 5. Relapse was established with a biopsy if possible or otherwise defined as radiological progression as per RECIST 1.1 [22]. In addition to using the established LoB for the combined four ctDNA markers, we used Receiver-Operating Characteristic (ROC) curves to evaluate the discriminative capabilities of the different biomarkers measured at baseline in detecting patients who had a relapse within 12 months. Area under the curve (AUC) values were reported for the individual markers and their combinations. Logistic regression models were used to estimate the probability of relapse as investigated in the multivariate ROC analyses.

Final data analysis was performed using STATA ver. 18 (StataCorp, College Station, TX, USA).

## Results

Recruitment for this study began in December 2019. By the inclusion cut-off date of September 25, 2023, 74 patients had been included in the study. The date of final follow-up was May 11, 2025, and all patients had at least 12-month follow-up at this timepoint.

**Fig. 1:**
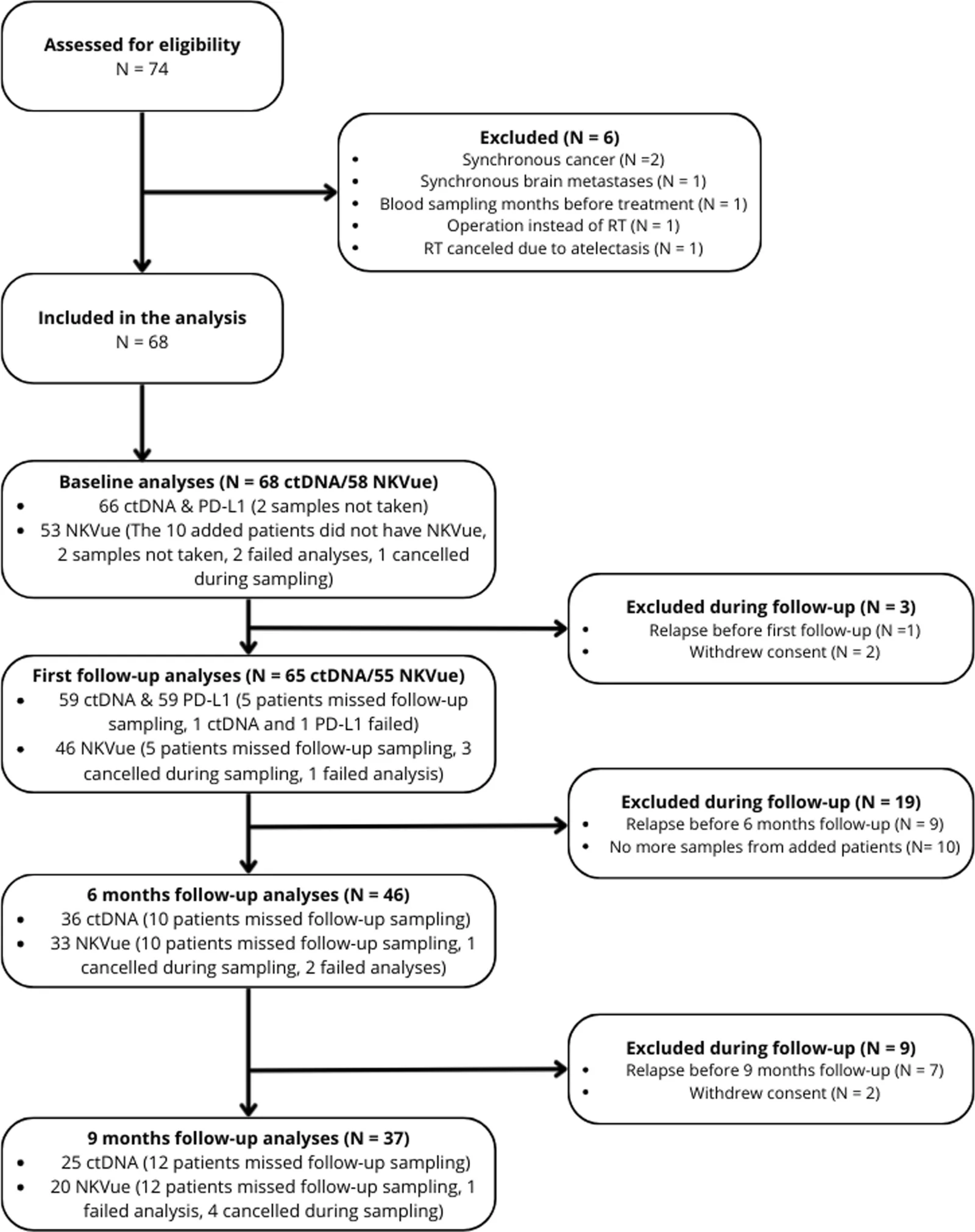
Inclusion and analysis CONSORT diagram

Figure 1 shows a detailed flowchart of the patient inclusion and blood sampling.

Two patients did not have baseline blood samples taken, but contributed with later blood samples to the study and remained in the cohort.

Nine patients did not have blood sampling performed at the first follow-up visit following completion of RT. We used the blood sample taken at the end of treatment instead, which explains the short duration between the first and second blood samples for some SBRT patients.

Soluble PD-L1 analyses were not available for 6- and 9-month control blood samples.

Table 1 shows the patient characteristics. Two patients were treated with SBRT with 50 Gy in five fractions because of proximity to the main bronchi. All patients receiving long-course RT also received concomitant chemotherapy, with carboplatin + vinorelbine being most predominant.

**Table 1:** Patient and treatment characteristics

Patient and treatment characteristics	Value, *n* (%)
Total number of patients	68
Age, median (range and IQR)	70 years (52–86 years, IQR 11 years)
Male sex	36 (53%)
Histologic subtype	
Adenocarcinoma	37 (54%)
Squamous cell carcinoma	25 (37%)
Other^a^	6 (9%)
Disease stage	
I	32 (47%)
II	3 (4%)
III	33 (49%)
Radiation treatment	
Long-course RT (24–33 fractions)	35 (51%)
SBRT (3–5 fractions)	33 (49%)
Concomitant chemotherapy (*N* = 35 (51%))	
Cisplatin + vinorelbine	6 (17%)
Carboplatin + vinorelbine	26 (74%)
Vinorelbine monotherapy	3 (9%)
WHO performance status	
0	13 (19%)
1	40 (59%)
2	15 (22%)
Smoking status	
Never	1 (2%)
Former	34 (50%)
Active	33 (49%)
Smoking—number of pack years, median (IQR)	41 pack years (IQR 18)
Body mass index, median (IQR)	25 kg/m^2^ (IQR 6)
Charlson’s comorbidity index, median (range and IQR)	6 (3–10, IQR 1)
Adjuvant Durvalumab following RT	11 (16%)
Median number of series received (range and IQR)	8 series (5–12, IQR 7)

^a^Comprised adenosquamous carcinoma (*N* = 1), poorly differentiated non-small cell carcinoma (*N* = 2), and non-small cell carcinoma not otherwise specified (*N* = 3). *IQR* Interquartile range;

### Relapse pattern following curative RT for lung cancer

In the cohort, 26 out of the 68 patients (38%) experienced a relapse within 12-month follow-up. Fourteen of these relapses were verified with a biopsy; the remaining twelve were radiological relapses. Six of the relapsed patients died before 12-month follow-up.

Twenty-one out of 36 patients with stage II–III disease relapsed during the first 12 months (58%), whereas only 5 out of 32 stage I patients relapsed in the same time period (16%).

### NKA and sPD-L1 analyses and risk of relapse within 12-month follow-up

We evaluated the association between NKA status (normal vs. reduced) at the different blood-sampling timepoints and the risk of relapse within 12 months of follow-up (Table 2). Twenty one of the 26 patients with a relapse within 12 months had baseline NKA measurements, and 2/3 of these were reduced at baseline. The Pearson chi^2^ test revealed a significantly higher risk of experiencing a relapse within 12 months after curative RT for patients with reduced NKA at baseline (*p* = 0.01).

**Table 2:** NKA levels and the risk of relapse within 12-month follow-up

Blood sampling timepoint	NKA levels—cutoff 250 pg/mL	Patients without relapse < 12 months follow-up, *n* (%)	Patients with relapse < 12 months follow-up, *n* (%)	Pearson chi^2^
Baseline	Normal	22 (42%)	7 (13%)	*p* = 0.01 *N* = 53
	Reduced	10 (19%)	14 (26%)	
First control (5 weeks or 3 months)	Normal	23 (50%)	7 (15%)	*p* = 0.31 *N* = 46
	Reduced	10 (22%)	6 (13%)	
Six months	Normal	13 (39%)	1 (3%)	*p* = 0.10 *N* = 33
	Reduced	12 (36%)	7 (21%)	
Nine months	Normal	11 (55%)	0 (0%)	*p* = 0.19 *N* = 20
	Reduced	7 (35%)	2 (10%)	

We also compared median sPD-L1 levels between patients who relapsed within 12 months of follow-up and those who did not, as shown in Table 3. There was a trend toward higher baseline sPD-L1 in patients who relapsed within 12 months of follow-up (*p* = 0.052), compared to those who did not relapse.

**Table 3:** sPD-L1 levels and the risk of relapse

Blood sampling timepoint	Soluble PD-L1 levels—pg/mL	Did not relapse < 12 months follow-up	Did relapse < 12 months follow-up	Total, *N*	Wilcoxon rank sum
Baseline	Patients, *n*	40 patients	26 patients	66 patients	*p* = 0.05
	Median (IQR)	69.5 (34.5)	85 (54)	75.5 (40)	
First control	Patients, *n*	39 patients	20 patients	59 patients	*p* = 0.31
	Median (IQR)	75 (29)	82.5 (62.5)	76 (36)	

### ctDNA status and risk of relapse within 12-month follow-up

Twenty-seven patients had positive baseline samples. This dropped to eight patients at the first control sampling (two of those were not positive at baseline). Fourteen of the twenty-one relapsing stage II–III patients had positive ctDNA status in the last blood sample before the relapse. In contrast, none of the five stage I relapsing patients had any positive ctDNA measurements.

The association between ctDNA status (negative vs. positive) at the different blood-sampling timepoints and the risk of relapse within 12 months of follow-up was also evaluated (Table 4). Patients with a positive ctDNA status at baseline (*p* = 0.03) or at 6 months (*p* < 0.01) had a significantly higher risk of relapse within 12 months.

**Table 4:** ctDNA status and the risk of recurrence within 12-month follow-up

Blood sampling timepoint	ctDNA status	Patients without relapse < 12 months follow-up, *n* (%)	Patients with relapse < 12 months follow-up, *n* (%)	Pearson chi^2^
Baseline	Negative	28 (42%)	11 (17%)	*p* = 0.03 *N* = 66
	Positive	12 (18%)	15 (23%)	
First control (5 weeks or 3 months)	Negative	35 (59%)	16 (27%)	*p* = 0.70 *N* = 59
	Positive	5 (8%)	3 (5%)	
Six months	Negative	26 (72%)	2 (6%)	*p* < 0.01 *N* = 36
	Positive	2 (6%)	6 (17%)	
Nine months	Negative	20 (80%)	2 (8%)	*p* = 0.33 *N* = 25
	Positive	2 (8%)	1 (4%)	

### Baseline ROC analyses for the risk of relapse within the first 12 months following treatment

**Fig. 2:**
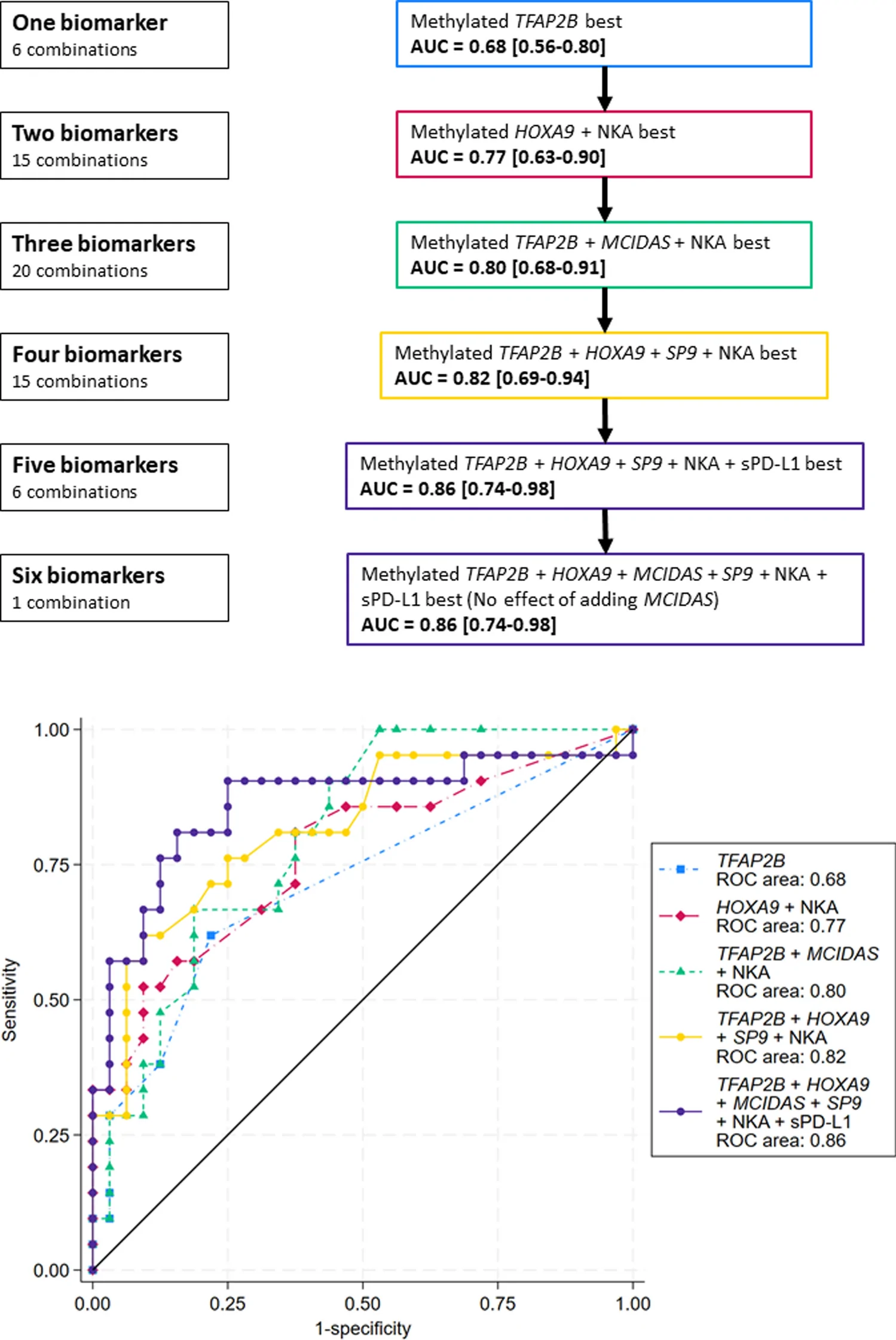
Stepwise ROC analysis of baseline biomarkers and the risk of relapse within 12-month follow-up

We performed ROC analyses of the four methylated ctDNA markers, sPD-L1 values, and NKA at baseline prior to RT to assess their ability to discriminate patients who relapsed within 12 months of follow-up. The analyses were done in a stepwise fashion (Fig. 2).

The discriminative value for each of the four methylated ctDNA markers at baseline was modest, with *MCIDAS* AUC value 0.65 [95% CI 0.51–0.78], *SP9* AUC 0.66 [0.53–0.79], *HOXA9* AUC 0.68 [0.54–0.82], and *TFAP2B* AUC 0.68 [0.56–0.80]. The discriminative value for the binary NKA marker had an equivalent AUC value of 0.68 [95% CI 0.55–0.81]. The continuous sPD-L1 marker had a likewise modest AUC of 0.64 [95% CI 0.50–0.78].

When combining two markers, AUC rose to 0.77 [95% CI 0.63–0.90] for the combination of *HOXA9* and NKA. The addition of extra markers consistently increased the AUC. As seen in Fig. 2, all four methylated ctDNA markers contributed a little to the increase in AUC. However, with five or six markers, there was no difference in the AUC whether *MCIDAS* was included or not—the AUC was 0.86 [95% CI 0.74–0.98] in both cases. Using DeLongs test to compare the AUC of 1 marker (*TFAP2B*) with the AUC of 6 markers, the obtained *p* value was 0.068.

Between 6- and 12-month follow-up, only 8 out of 36 sampled patients had a relapse, so the data material was too small to allow meaningful ROC analyses.

**Fig. 3:**
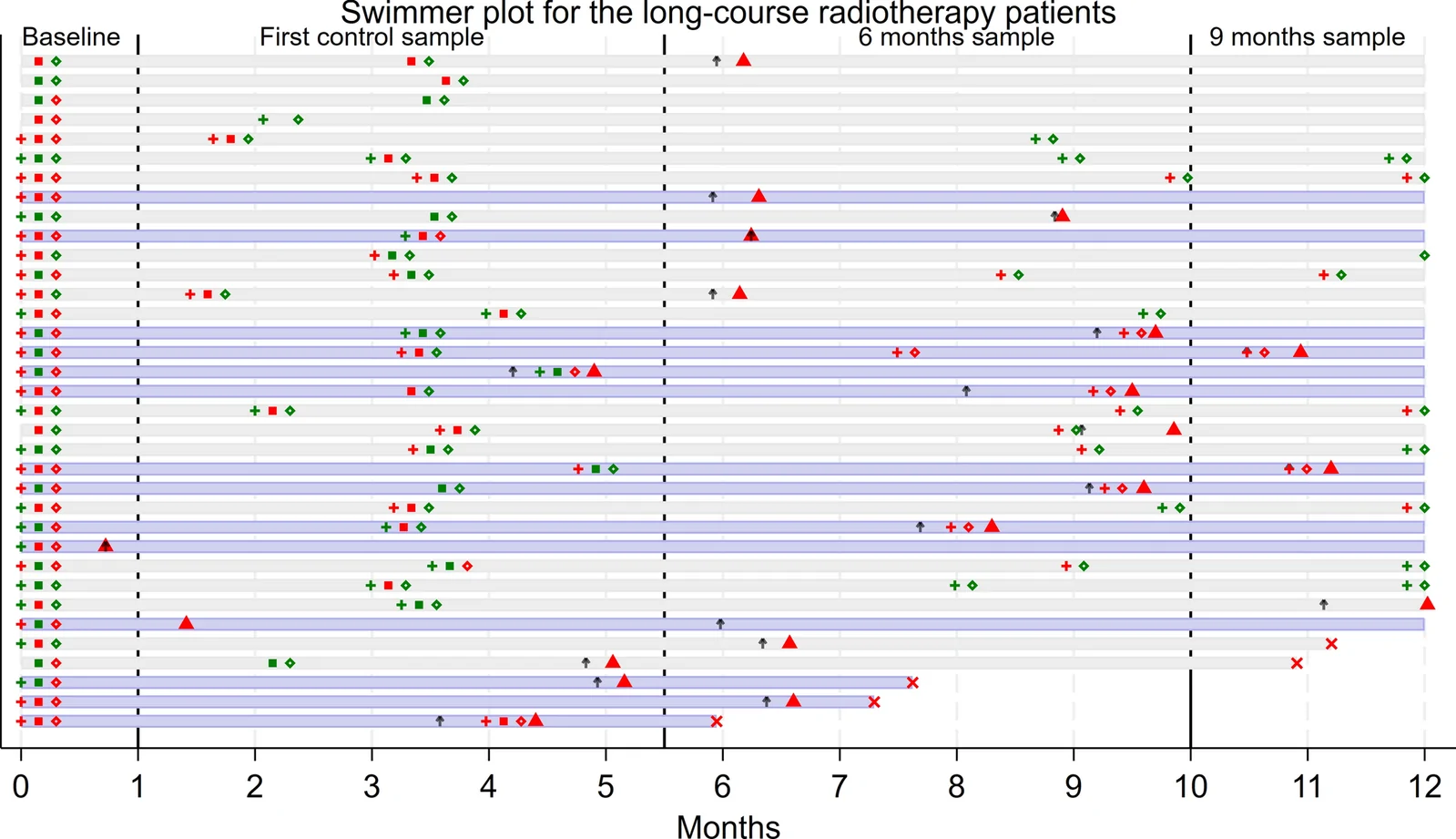
Swimmer plot with patients receiving long-course RT. *NKA* Natural Killer-cell activity. *PD-L1* Programmed death-ligand 1. *ctDNA* Circulating tumor DNA

Figure 3 shows a swimmer plot with timing and results of blood samples, relapse, and death for each of the patients treated with long-course RT. See Online Resource A2 for the same plot with the patients treated with SBRT. The time of relapse is the date of the CT scan revealing the relapse. As the patients often had the blood sampling performed during the hospital visit where they received their CT-scan results, this explains why their relapse came a short time before the final blood sampling.

## Discussion

The use of blood based biomarkers in lung cancer has been widely studied in recent years; however, not many studies concern the use of biomarkers in patients treated with RT. Given that a substantial number of patients with localized lung cancer are treated primarily with RT, we saw a demand for the present study, which to our knowledge is the first concerning methylated ctDNA, sPD-L1, and NKA in patients with localized or locally advanced NSCLC treated with curative RT.

This study shows the statistically significant discriminative potential of NKA, sPD-L1, and methylated ctDNA predicting the risk of relapse during 12-month follow-up after curative RT for lung cancer.

NKA measured at baseline was prognostic by itself, meaning that patients with a baseline value of NKA below 250 pg/mL had a statistically significant higher risk of experiencing a relapse within a 12-month follow-up period. This is in line with the findings of Choi et al. [23], who found a significant correlation between reduced baseline NKA levels and shorter progression-free survival (PFS) in a cohort of 34 patients with advanced NSCLC treated with immune checkpoint inhibitors (ICIs) (median PFS 37 vs. 78 days, respectively; log-rank test *p* = 0.003). They likewise found that reduced baseline NKA levels predicted a higher risk of progressive disease (PD) at the follow-up response assessments, thus predicting a worse outcome of the treatment. Worth mentioning is that this study used a higher cutoff of 1200 pg/mL NKA, dividing patients into the normal and reduced groups, obtained through an ROC analysis of their data, instead of the cutoff of 250 pg/mL recommended by the manufacturer and used in our study.

Having a positive ctDNA status, i.e., two or more ctDNA markers above the LoB at baseline, was also statistically significantly prognostic of a higher risk of experiencing a relapse during the 12-month follow-up. This was also demonstrated in a study involving 132 patients with advanced lung cancer by Frank et al. [24], who showed a significantly longer OS in patients who had undetectable ctDNA at baseline (median 578 vs. 296 days, respectively; HR 0.66 [0.44–0.98]. This study used a more extensive, tumor-informed approach where every patient underwent a tissue biopsy, which was tested with the TSO500 HT gene panel (Illumina). The identified mutations were then used to design a personalized ctDNA assay, measured in plasma. In contrast, our study used a less labor-intensive, tumor-agnostic approach that may be easier to implement in clinical practice.

We also found that having positive ctDNA status at the 6-month blood sampling was significantly prognostic of developing a relapse within the first 12 months. However, due to the small numbers in this analysis (*N* = 36 and only 8 relapses), we tend to regard this as a random result, although the same association was found in another study examining mutations in ctDNA after treatment of lung cancer with chemo-RT [25]. In this study, the detection of ctDNA 4.5 months after treatment was significantly associated with higher odds for tumor recurrence (OR 5.4 [1.1–31]), where ctDNA was detectable in 16 patients out of 39.

The baseline sPD-L1 value was borderline significant by itself, meaning that patients with values above the median may have a higher risk of experiencing a relapse before 12-month follow-up compared to patients with values below the median. Use of a more optimal cutoff for sPD-L1 could potentially have resulted in a more desirable outcome; however, this limit is not known. A study by Mazzaschi and colleagues illustrates the application of an alternative cutoff and an alternative outcome [26]. Using the same immunoassay as in our study, they quantified sPD-L1 in baseline samples from 109 patients with advanced lung cancer. A cutoff of 113 pg/mL, defined by CART tree analysis, was applied. They found that patients with sPD-L1 levels above this cutoff exhibited a significantly shorter median PFS (3.8 months) than those with sPD-L1 levels below the cutoff (11.9 months—HR 2.55 [1.50–4.32]). Like us, they also hypothesized that NK cells could be of prognostic value in lung cancer. While their results showed that patients with low NK-cell count had shorter PFS (median PFS 3.8 months vs. 10.2 months, respectively, *p* < 0.001), our study demonstrated that lung cancer patients with low NKA experienced shorter PFS. The two different approaches preclude a direct comparison of the results. NK-cell count has been shown to be a relevant biomarker in some conditions; however, as this cell type can be affected by a systemic immunosuppressive environment, they may be present, but not functional. The methodology used in our study aimed to mitigate the impact of such bias. The findings from both studies, however, point to NK cells as a prognostic biomarker in lung cancer, highlighting their possible utility in guiding clinical decision-making.

The ROC analysis of the four methylated ctDNA loci also revealed some discriminative potential; however, the highest AUC values required the addition of NKA and sPD-L1 in the ROC analysis.

Most of the patients with reduced baseline NKA, increased sPD-L1, and/or presence of methylated ctDNA were in stage 3 and received long-course RT. Compared to the patients treated with SBRT, they had extensive disease with larger primary tumors and/or involvement of regional lymph nodes. These factors might have increased the shedding of ctDNA and sPD-L1 to the circulation, and increased the risk of developing reduced NKA [27]. The stage III patients received chemo-RT, a less-effective treatment than SBRT, and have a higher risk of relapse due to a more advanced disease with a larger disease burden. Still, it may seem that having a reduced baseline NKA level or positive methylated ctDNA status could be a warning that intensified treatment and closer follow-up are indicated for stage III patients. This could mean receiving adjuvant immunotherapy if eligible and/or doing follow-up CT scans at shorter intervals.

The values of these biomarkers for stage I lung cancer seem modest and require further investigation, as none of the relapsing patients were positive for ctDNA, and only two out of seven patients with reduced NKA at baseline experienced a relapse. The ambiguous ability of ctDNA to distinguish relapsing patients from non-relapsing patients was also shown in a subgroup analysis of 13 patients with NSCLC treated with curative radiotherapy or chemoradiotherapy in the Danish SUPE_R trial [28]. In this subgroup analysis, having detectable mutated ctDNA either at an early follow-up < 4.5 months following treatment or at a later follow-up > 4.5 months after treatment was not associated with higher risk of recurrence within 24 months after RT. Although only a subgroup analysis with a small sample size, this supports the need for larger studies with more patients and longer follow-up to fully establish the role of ctDNA and other biomarkers in detection of relapse and minimal residual disease in stage I lung cancer patients.

Several patients had PET/CT scans made during their follow-up program due to enrollment in other studies, but we did not see a higher relapse rate among these patients compared to the patients followed with CT scans, so we do not expect this to have influenced our results.

At the first control, between 5 weeks and 3 months after the treatment, most patients had regained normal levels of the biomarkers of interest, i.e., ctDNA was below the cutoff; levels of sPD-L1 were below the median of the baseline samples, and NKA was no longer reduced. This is in line with the above-mentioned study examining biomarkers after RT for lung cancer, where blood sampled on average 1.6 months after RT did not discriminate patients with later relapse [25]. This might be explained by the cancer cells being eliminated and not shedding measurable amounts of ctDNA shortly after completion of RT. With more months passing, the growing tumor might again shed measurable amounts of ctDNA, increased sPD-L1, or cause reduced NKA. However, our study did not have enough relapses between 6 and 12 months following RT to allow meaningful analyses of these possible patterns.

The strengths of this study include the detailed panel of blood analyses being performed. The analyses are tumor-agnostic and not very resource-demanding, allowing easy implementation in clinical practice. The cohort was also well described and had complete radiologic and clinical follow-up in the period of interest.

The limitations of this study include the limited follow-up period of 12 months following RT, the number of patients with missing samples, and the fact that the cohort is a combination of patients treated with SBRT and long-course RT. Nevertheless, this study presents interesting data suggesting the prognostic capability of the combination of ctDNA, sPD-L1, and NKA.

## Conclusion

This study highlights the ability of tumor-agnostic methylated ctDNA, sPD-L1, and NKA measured at baseline in predicting the risk of recurrence during 12 months after curative RT for localized and locally advanced NSCLC.

## Supplementary Information

Below is the link to the electronic supplementary material.

Supplementary file1 (DOCX 263 KB)

## Data Availability

The data material related to this article is currently unavailable, as follow-up for all patients is not complete. The data material will be available at a later time after written request to the corresponding author.

## Abbreviations

AUC: Area under the curve
CART: Classification and regression tree
CI: Confidence interval
CT: Computed Tomography
ctDNA: Circulating tumor deoxyribonucleic acid
ddPCR: Droplet digital polymerase chain reaction
EDTA: Ethylenediaminetetraacedic acid
ELISA: Enzyme-linked immunosorbent assay
GEO: Gene expression omnibus
Gy: Gray
*HOXA9*: Homeobox A9
IFN-γ: Interferon gamma
IQR: Interquartile range
LoB: Limit of blank
LoD: Limit of Detection
*MCIDAS*: Multiciliate differentiation and DNA synthesis associated cell cycle protein
NKA: Natural Killer-cell activity
NSCLC: Non-small cell lung cancer
OR: Odds ratio
PET: Positron emission tomography
PFS: Progression-free survival
PS: Performance status
QQ-plot: Quantile–quantile plot
RECIST: Response evaluation criteria in solid tumors
ROC: Receiver-operating characteristics
RT: Radiotherapy
SBRT: Stereotactic body radiation therapy
*SP9*: Transcription factor SP9
sPD-L1: Soluble programmed death-ligand 1
TCGA: The cancer genome atlas
*TFAP2B*: Transcription factor AP-2 beta
