# Supplementary material for: The prognostic value of methylated ctDNA, soluble PD-L1, and NK-cell activity on the risk of relapse after curative radiotherapy of non-small cell lung cancer
Source: Clin Transl Oncol. 2026 Mar 16;28(9):3790–802. doi: 10.1007/s12094-026-04317-5 (PMC13499851; doi:10.1007/s12094-026-04317-5)
Supplement: Supplementary file 1 [file 12094_2026_4317_MOESM1_ESM.docx]

**Supplementary material – extra figures and a special case**

## **The prognostic value of methylated ctDNA, soluble PD-L1, and NK-cell activity on the risk of relapse after curative radiotherapy of non-small cell lung cancer**

## Authors and affiliations

Thomas Leth Fink ^a,b,*^<https://orcid.org/0000-0002-3363-7492>, Rikke Fredslund Andersen ^a,c^<https://orcid.org/0000-0002-5894-7745>, Cecilie Mondrup Jacobsen ^a,b,c^<https://orcid.org/0000-0003-3238-3889>, Line Nederby ^a,b,c^<https://orcid.org/0000-0002-0101-328X>, Mads Malik Aagaard Jørgensen ^d^<https://orcid.org/0000-0003-4742-7047>, Charlotte Kristiansen ^a^<https://orcid.org/0000-0002-8073-5781>, Torben Schjødt Hansen ^a^<https://orcid.org/0009-0008-6250-0242>, Sara Witting Christensen Wen ^b,c^<https://orcid.org/0000-0003-3755-0922>, Christa Haugaard Nyhus ^a^, Rune Slot Thing ^a^<https://orcid.org/0000-0002-2683-2847>, Signe Timm ^a,b^<https://orcid.org/0000-0002-5862-7784> and Torben Frøstrup Hansen ^a,b^<https://orcid.org/0000-0001-7476-671X>,

^a^ Department of Oncology, Lillebaelt Hospital, Vejle, University Hospital of Southern Denmark, 7100 Vejle, Denmark

^b^ Institute for Regional Health Research, University of Southern Denmark, Odense M, Denmark

^c^ Department of Biochemistry and Immunology, Lillebaelt Hospital, Vejle, University Hospital of Southern Denmark, 7100 Vejle, Denmark

^d^ Department of Clinical Genetics, Lillebaelt Hospital, Vejle, University Hospital of Southern Denmark, 7100 Vejle, Denmark

*: Corresponding author: tlf@rsyd.dk - Tel.: +45 29649145 – Department of Oncology, Lillebaelt Hospital – University Hospital of Southern Denmark, Vejle DK-7100, Denmark

Supplementary figure A1 – box plots of PD-L1 levels at baseline and first control.

sPD-L1: Soluble programmed death ligand 1.

Supplementary figure A2 – Swimmer plot for the SBRT patients

SBRT: Stereotactic body radiation therapy. NKA: Natural Killer cell activity. PD-L1: Programmed death ligand 1. ctDNA: Circulating tumor DNA.

Supplement A3.

*A special case*

One male patient aged 78, who was treated with SBRT for an apical T1c-tumor, showed a consistently high number of positive droplets when measuring methylated ctDNA from baseline and onwards during his follow-up CT scans, without any sign of relapse. We have not been able to identify why his methylated ctDNA levels are persistently positive. He is known with focal non-motoric epilepsy, treated with Lamotrigine, and previous ST-elevated myocardial infarction and transcatheter aortic valve implantation following aortic stenosis. Droplet count in all samples and all four methylations was between 5 and 98. His baseline and first control NKA were normal, but below normal at 6 months. The baseline sPD-L1 was quite high (166 pg/mL) but dropped to 96 at first control, which is still above the median of the cohort.
